# Secretan’s Syndrome of the Hand: Literature Review and Surgical Case Report of a Rarely Documented Condition

**DOI:** 10.3390/jpm15120586

**Published:** 2025-12-01

**Authors:** Andrea Cruciani, Emanuele Gerace, Gianmarco Vavalle, Elisa Di Dio, Silvia Pietramala, Lorenzo Rocchi

**Affiliations:** 1 Department of Orthopaedics and Traumatology, Fondazione Policlinico Universitario A. Gemelli IRCCS, 00168 Rome, Italy; andrea.cruciani01@icatt.it (A.C.); gianmarco.vavalle01@icatt.it (G.V.); elisa.didio01@icatt.it (E.D.D.); silvia.pietramala01@icatt.it (S.P.); 2 Università Cattolica del Sacro Cuore, Largo Francesco Vito, 1, 00168 Rome, Italy; lorenzo.rocchi@policlinicogemelli.it; 3 Hand Surgery and Orthopedics Unit, Department of Orthopaedics and Traumatology, Fondazione Policlinico Universitario A. Gemelli IRCCS, 00168 Rome, Italy

**Keywords:** case study, chronic indurated edema, differential diagnosis, dorsal hand edema, hand surgery, Secretan’s syndrome

## Abstract

**Background:** Secretan’s syndrome is a rare and under-recognized condition characterized by chronic, indurated, non-pitting edema of the dorsal hand with thumb sparing. Twelve studies reporting 17 patients have been published worldwide, mostly as isolated case reports, and its pathogenesis remains debated between traumatic, inflammatory, and factitious mechanisms. This article presents a surgically managed hyperplastic case and a literature review, highlighting how precision medicine principles can guide diagnosis and treatment. **Materials and Methods:** A 36-year-old healthcare worker developed progressive dorsal swelling of the left hand following minor trauma, with marked restriction of metacarpophalangeal flexion. Laboratory tests and radiographs were normal. MRI demonstrated peritendinous fibrosis encasing the extensor tendons. Psychiatric evaluation excluded factitious behavior. Due to functional limitation and MRI evidence of fibrosis, selective fasciotomies and tenolysis were performed. A systematic literature review was conducted, in accordance with the PRISMA 2020 guidelines, to summarize epidemiology, clinical and imaging features, histopathology, and management options. **Results:** Histology revealed fibro-adipose tissue with chronic inflammatory changes and CD68+ histiocytic aggregates; microbiological cultures were negative. Postoperative rehabilitation enabled significant functional recovery. The literature review confirmed the scarcity of published cases and the absence of standardized guidelines. MRI proved the most informative imaging tool, while surgery was described only in refractory forms. **Conclusions:** This case and review illustrate how a precision medicine approach can optimize management of rare disorders. Early MRI-based diagnosis, multidisciplinary assessment, and phenotype-driven surgical intervention allowed tailored treatment and favorable outcome. Personalized care that integrates clinical features, imaging findings, and patient-specific factors may improve results despite the limited evidence base.

## 1. Introduction

Secretan’s syndrome, first described by Henri-François Secretan in 1901, is a rare and often under-recognized condition defined by persistent, non-pitting, indurated edema of the dorsal hand after minor trauma [1]. Classically, swelling predominates over the metacarpal region with sparing of the thumb and may cause finger flexion limitation and prolonged functional impairment [2,3]. Early reports in Swiss workers highlighted the chronicity of the edema and raised the possibility of secondary gain in selected contexts [3,4]. Histology typically shows fibrotic induration and peritendinous fibrosis, albeit non-specific findings [1,5]. Clinically, the course ranges from spontaneous resolution to a hyperplastic, fibrotic form that may require surgical management [2,6]. Imaging contributes to characterization: radiographs are often unremarkable, whereas MRI depicts peritendinous edema and fibrotic involvement of the extensor compartments [7]. Pathogenesis remains debated. Proposed mechanisms include post-traumatic/inflammatory pathways akin to lymphedema or reflex sympathetic dystrophy, and a factitious component associated with self-inflicted injury or tight bandaging; psychiatric comorbidity has been described in atypical presentations [8,9,10]. The differential diagnosis is broad and includes complex regional pain syndrome (CRPS), lymphedema, superficial venous thrombosis, cellulitis, fasciitis, and extensor tenosynovitis. In contrast to CRPS, which according to the Budapest criteria is typically associated with disproportionate burning pain, vasomotor or sudomotor alterations, and trophic changes, Secretan’s syndrome presents with firm, non-pitting dorsal edema, thumb sparing, and absence of autonomic or trophic disturbances. Although the hand is the typical site, rare cases involve the foot and ankle [5]. More recently, studies on chronic hand edema have emphasized the role of high-resolution MRI and ultrasound elastography to better characterize peri-tendinous fibrosis and distinguish it from inflammatory or vascular conditions [11,12]. Recent reviews call for greater awareness and more standardized diagnostic criteria to guide conservative care and to identify candidates for surgery in hyperplastic forms [13,14]. Given the scarcity of published cases and the lack of standardized diagnostic criteria, there is a growing consensus on the need for greater awareness and multicenter collaboration to define optimal management strategies [15].

We report a surgically confirmed case of Secretan’s syndrome of the hand and critically appraise the literature to outline presentation, imaging and histology, differential diagnosis, and management.

## 2. Materials and Methods of Literature Review

A structured literature search was conducted in PubMed, Embase, and Scopus up to March 2025, in accordance with the Preferred Reporting Items for Systematic Reviews and Meta-Analyses (PRISMA) 2020 guidelines [16]. The search terms included “Secretan’s syndrome”, “factitious lymphedema”, “chronic dorsal hand edema”, and “peritendinous fibrosis”. The complete search strings for each database are reported in Supplementary Materials (Table S1) to ensure transparency and reproducibility. We included case reports, case series, and reviews that described clinical presentation, imaging, histology, treatment, or outcome of patients with Secretan’s syndrome. Publications in English, French, and Italian were considered. Articles lacking sufficient clinical detail or reporting unrelated conditions were excluded. The reference lists of selected papers were screened to identify additional relevant reports. Title and abstract screening, as well as full-text eligibility assessment, were independently performed by two reviewers, with disagreements resolved through discussion. Data extraction was performed by one reviewer and cross-checked by a second to minimize errors. In total, 95 records were identified; after removing 13 duplicates, 82 records were screened. 62 were excluded at the title/abstract level. 20 full-text articles were assessed, with 8 excluded (5 for insufficient clinical details and 3 for unrelated conditions). 12 studies met inclusion criteria and were included in the review. The study selection process is summarized in the PRISMA 2020 flow diagram in Supplementary Materials (Figure S1), and the completed PRISMA 2020 checklist is also provided in Supplementary Materials (Table S2). Given the rarity of the condition and the limited number of studies available, no protocol was registered on PROSPERO.

## 3. Review of the Literature

Secretan’s syndrome was first described in 1901 and remains one of the rarest entities in hand surgery. Overall, 12 studies reporting 17 patients have been published, predominantly from Europe. Most publications consist of isolated case reports, with only a handful of small series available. This scarcity of data explains why the condition is still poorly recognized, often misdiagnosed, and lacks standardized diagnostic criteria.

### 3.1. Epidemiology

The condition predominantly affects middle-aged adults, with no clear sex predilection. Occupational or repetitive microtrauma has been reported as a common trigger, though factitious etiology has been documented in several cases. While earlier reports emphasized traumatic or occupational factors, more recent literature suggests that psychiatric comorbidity and self-inflicted mechanisms may be underdiagnosed contributors. The limited number of cases precludes estimation of true incidence or prevalence.

### 3.2. Clinical Presentation

The hallmark is chronic, indurated, non-pitting edema on the dorsum of the hand, typically sparing the thumb. Limitation of MCP joint motion, especially flexion, is frequent. Pain and functional impairment are variably reported. Most cases describe a dull or pressure-like pain, sometimes present at rest but usually aggravated by motion, in contrast with the burning, disproportionate pain typical of CRPS. Other features such as skin color or temperature changes, sudomotor alterations, hypertrichosis, or nail dystrophy are usually absent, which further differentiates Secretan’s syndrome from CRPS and lymphedema.

### 3.3. Imaging

Radiographs are usually normal. MRI is consistently identified as the most useful diagnostic tool, demonstrating peritendinous edema, fibrotic bands, and fascial thickening. These findings help distinguish Secretan’s syndrome from cellulitis, venous thrombosis, and CRPS. Recent reports underline the added value of high-frequency ultrasound and advanced MRI sequences (diffusion-weighted or elastography-based), which may detect subtle peritendinous fibrosis earlier and better delineate tendon involvement. Ultrasound has also been suggested as a bedside tool to rule out vascular causes of dorsal hand edema.

### 3.4. Histology

Reported histology is non-specific, with fibrous tissue, inflammatory infiltrates, and peritendinous fibrosis. No pathognomonic pattern has been defined. Nevertheless, the presence of hyperplastic fibrotic tissue with CD68+ histiocytic aggregates has been described in some recent cases, supporting the hypothesis of a chronic inflammatory component.

### 3.5. Management

Treatment strategies vary widely. Many cases resolve with conservative measures such as compression, rest, and physiotherapy. Psychiatric evaluation is strongly recommended when factitious etiology is suspected. In more recent literature, a multidisciplinary approach combining rehabilitation, psychological support, and imaging-guided physiotherapy has been emphasized to prevent functional decline. Early mobilization and tailored hand therapy protocols are considered beneficial. Surgical intervention is rarely described and generally reserved for hyperplastic, refractory cases with functional compromise. Outcomes after surgery are variable but often favorable when fibrosis and adhesions are clearly documented. However, no standardized criteria exist for surgical indication, and the decision remains case-dependent.

A summary of the reported cases in the literature is presented in Table 1.

## 4. Detailed Case Description

### 4.1. Patient Information

A 36-year-old female healthcare worker (employed in a nursing home) presented with progressive dorsal swelling of the left hand following a minor closed contusion of the dorsum of the hand, without skin lacerations or open injury, sustained during work activities. She had no relevant medical history, no psychiatric disorders, and no chronic medication use.

### 4.2. Clinical Findings

**Figure 1:**
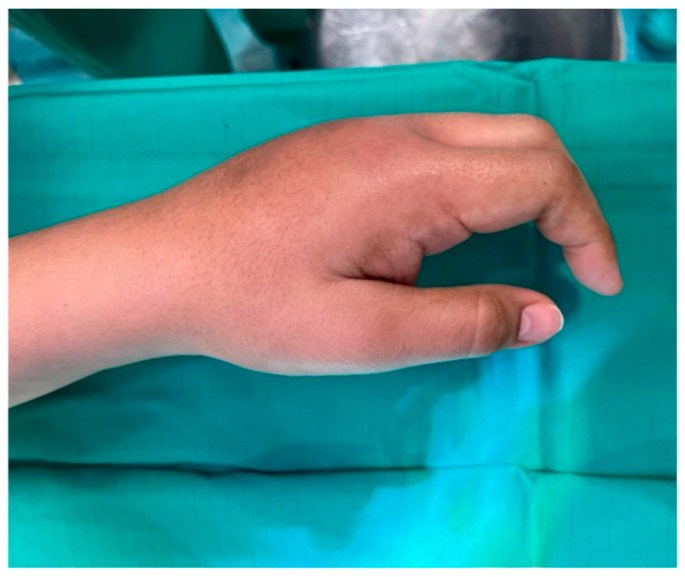
Preoperative documentation. Dorsal clinical view of the left hand showing firm, non-pitting edema with thumb sparing.

Examination revealed a firm, indurated, non-pitting edema over the dorsum of the hand with sparing of the thumb (Figure 1). Metacarpophalangeal (MCP) flexion of digits II–V was restricted to <20°, while extension was complete. The patient reported moderate, dull, chronic pain (VAS 7/10), present at rest without burning quality, radiating into the forearm, which worsened with movement. This was associated with significant limitations in both professional duties and daily activities. Grip strength was reduced on qualitative testing.

### 4.3. Diagnostic Assessment

**Figure 2:**
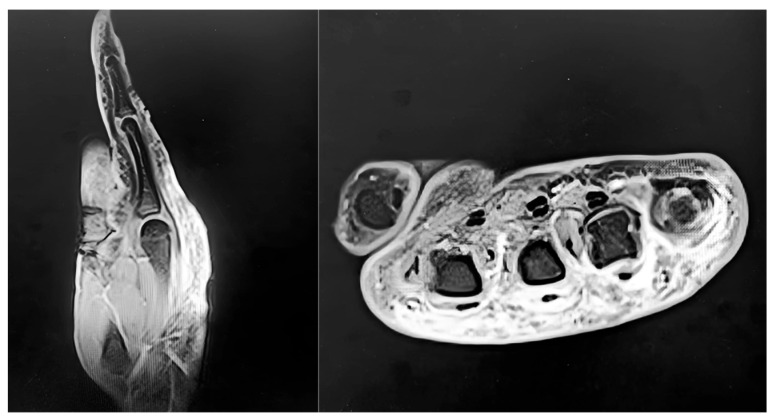
Preoperative MRI of the left hand showing peritendinous edema and a hypointense fibrotic band encasing the extensor tendons (compartments II–V).

Routine blood tests, erythrocyte sedimentation rate, and C-reactive protein were normal. The patient was afebrile. Radiographs showed no fractures, osteolysis, or arthropathy. Magnetic resonance imaging (MRI) of the left hand (3 February 2025) demonstrated peritendinous edema and a hypointense fibrotic band encasing the extensor tendons within compartments II–V (Figure 2). The fibrotic changes extended proximally up to anatomical zone VI but did not involve zone VII. The differential diagnosis included complex regional pain syndrome (CRPS), infectious or non-infectious extensor tenosynovitis, lymphedema, superficial venous thrombosis, cellulitis/fasciitis, and factitious injury. The combination of dorsal indurated non-pitting edema, thumb sparing, negative laboratory tests, and MRI findings was consistent with Secretan’s syndrome.

Given the literature describing possible factitious components, a psychiatric consultation was obtained both preoperatively and postoperatively with diagnosis of episodic mood disorder (ICD-9-CM code 296.90). No evidence of self-harm or active factitious behavior was identified, although psycho-educational support and follow-up were recommended to reinforce adherence to care.

### 4.4. Therapeutic Intervention

**Figure 3:**
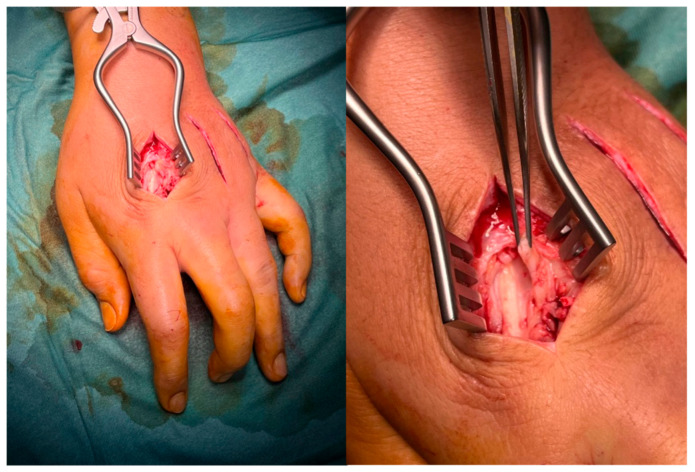
Intraoperative findings through dorsal approaches to intermetacarpal spaces II–IV, showing extensive peritendinous fibrosis and adhesions of the extensor tendons; selective fasciotomies and tenolysis were performed.

On 12 April 2025, surgery was performed under brachial plexus block in a day-surgery setting. A longitudinal dorsal incision was made across three intermetacarpal spaces (II–IV). Intraoperatively, extensive peritendinous fibrosis and adhesions of the extensor tendons were observed, without abscesses or purulent collections (Figure 3). The extensor compartments were not opened, as the fibrosis was confined to the peritendinous tissues without extension beyond zone VI. Selective fasciotomies and extensor tenolysis were carried out. Three tissue samples were collected for culture and histological examination. The wound was closed in layers, with a sterile dressing and functional bandage. Standard analgesia and early mobilization were prescribed.

### 4.5. Microbiological and Histological Findings

Cultures were negative. Histology (26 April 2025) showed fibro-adipose tissue with edema, chronic inflammation, amorphous material deposition, and aggregates of CD68+ histiocytic cells. These findings were non-specific but consistent with hyperplastic peritendinous fibrosis.

### 4.6. Follow-Up and Outcomes

**Figure 4:**
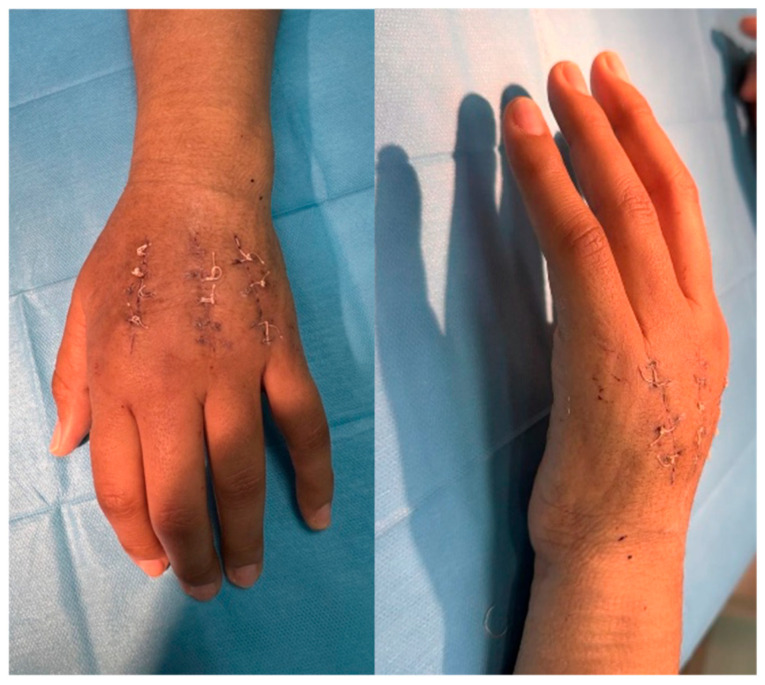
Early postoperative views. Anteroposterior and lateral aspect of the hand showing surgical incisions in healing phase, residual edema, and limited MCP flexion.

At 18 days postoperatively, MCP flexion was still limited to ~20° (Figure 4). At 4 weeks, motion improved to ~45° following initiation of physiotherapy, electrostimulation, and a home exercise program. At 3 months, extension was complete and flexion stabilized around 45°, with reduced pain and no recurrence of swelling. The patient resumed driving by 4 months. At 6 months, after structured rehabilitation and good compliance, MCP flexion reached ~70°, with marked reduction in pain and edema. No surgical or infectious complications occurred.

Functional outcomes at baseline, postoperative, and follow-up are summarized in Table 2.

## 5. Discussion

The present case demonstrates a classic clinical-radiological phenotype of Secretan’s syndrome: dorsal, indurated, non-pitting edema with thumb sparing, unremarkable laboratory workup, and MRI evidence of peritendinous fibrosis. Histology confirmed hyperplastic fibrotic tissue with CD68+ histiocytic aggregates, consistent with but not diagnostic for the syndrome.

Two aspects were crucial in this case: the early, imaging-based diagnosis prevented unnecessary empiric antibiotic therapies or prolonged diagnostic delays; and the multidisciplinary management, including psychiatric evaluation, was essential to exclude factitious behavior and to support adherence to treatment. It is also important to underline that the clinical presentation may easily mimic other conditions such as complex regional pain syndrome (CRPS) or lymphedema, which reinforces the need for careful differential diagnosis supported by imaging. In our patient, the diagnosis of CRPS was excluded based on the Budapest criteria. Pain was moderate (VAS 7/10), dull, and chronic, without burning quality or disproportion to the clinical findings. No vasomotor or sudomotor changes were observed, and the skin color and temperature were comparable to the contralateral side. There were no trophic alterations such as nail changes or hypertrichosis. Instead, the presence of firm, indurated, non-pitting edema with MRI-documented peritendinous fibrosis was consistent with Secretan’s syndrome and incompatible with a CRPS phenotype.

Surgical management was indicated due to extensive fibrosis and persistent functional limitation, aligning with the few previously reported cases requiring intervention. Functional recovery was substantial, with MCP flexion improving from <20° preoperatively to ~70° at 6 months.

When compared with the literature, our case aligns with Redfern et al. [3], who reported functional improvement after surgical release of peritendinous fibrosis. A similar role for surgery in refractory disease was noted by Abnousi and Chou [5], who described resolution following excision of fibrotic tissue in the foot. Conversely, most reports, such as those by Angelini et al. [1] and Collet et al. [4], describe conservative management, indicating that surgery should be considered only in selected cases. MRI has repeatedly been identified as the most informative diagnostic tool. In our patient it confirmed peritendinous fibrosis and guided surgical planning, consistent with the findings of Whitney and Jones [7]. Histological results, although non-specific, resembled those reported by Lemmens et al. [13], who described fibrotic changes without pathognomonic features.

The current literature, however, is limited by several critical aspects. First, the number of published cases remains extremely small, with most reports consisting of single-patient descriptions or very small series. This makes it impossible to perform meta-analyses or to establish evidence-based recommendations. Second, treatment strategies are markedly heterogeneous, ranging from conservative observation to psychiatric interventions, compression therapy, physiotherapy, and surgery, with no standardized criteria for patient selection or outcome evaluation. Third, outcome reporting is inconsistent: some studies provide detailed functional recovery metrics, whereas others only describe subjective improvement. Such heterogeneity limits comparability and weakens the strength of available evidence [11,12,15].

Recent advances in musculoskeletal imaging highlight a potential avenue for progress. High-resolution MRI and ultrasound elastography have been proposed as tools for better characterization of peritendinous fibrosis, allowing earlier differentiation from CRPS, cellulitis, or lymphedema [11]. Similarly, structured rehabilitation protocols integrating physiotherapy, electrostimulation, and occupational therapy are increasingly emphasized in chronic hand edema of various etiologies, suggesting potential benefit also in Secretan’s syndrome [12]. Furthermore, multidisciplinary management, including psychological support, has been underscored as essential in rare disorders where factitious or psychosomatic components may coexist [15].

Overall, this case highlights the value of a personalized, precision medicine approach: while conservative treatment remains first-line, surgery may be justified in hyperplastic forms with functional impairment, provided that imaging supports the diagnosis and psychiatric assessment excludes factitious causes.

Despite following PRISMA guidelines, this review is limited by the small number of available studies and their heterogeneity. Most reports consist of isolated case descriptions, with variable detail and limited outcome data, reducing the strength of evidence. Furthermore, the risk of bias of individual studies could not be systematically assessed, as the available evidence consists mainly of case reports and small case series.

In addition, the treatments reported in the literature are markedly heterogeneous, ranging from conservative measures and psychiatric interventions to surgical approaches, with no standardized criteria for patient selection or outcome reporting. This variability, combined with the absence of validated diagnostic guidelines, makes it difficult to establish clear recommendations for clinical practice. Consequently, the conclusions that can be drawn remain largely descriptive, and their generalizability is limited.

## 6. Conclusions

Secretan’s syndrome remains a diagnostic challenge due to its rarity and heterogeneous presentation. This case illustrates how principles of precision medicine can be applied: combining clinical phenotype, imaging findings, psychiatric evaluation, and tailored surgical management. In hyperplastic forms with refractory fibrosis, selective fasciotomies and tenolysis, followed by structured rehabilitation, can restore function and improve quality of life.

The available literature is highly heterogeneous, and the absence of standardized diagnostic or therapeutic guidelines limits the ability to provide evidence-based recommendations. Nevertheless, recent advances in imaging and rehabilitation suggest promising directions for future care. Multicenter collaborations and prospective registries are needed to clarify the natural history, refine diagnostic criteria, and define the role of surgery versus conservative treatment.

## Figures and Tables

**Table 1:** Reported cases of Secretan’s syndrome in the literature.

Author, Year	No. of Cases	Site	Diagnostic Findings	Management	Outcome
Grobmyer et al., 1968 [10]	1	Hand	Indurated dorsal edema	Surgical (closed lymphangioplasty)	Persistent edema
Fleming, 1977 [7]	1	Hand	Indurated dorsal edema, fibrosis (histology)	Conservative	Chronic course with persistent symptoms
Angelini et al., 1982 [1]	1	Hand	Non-pitting dorsal edema; fibrosis (histology)	Conservative	Partial resolution
Redfern et al., 1982 [9]	4	Hand	Peritendinous fibrosis	Surgical release	Functional improvement
Winkelmann & Barker, 1990 [11]	2	Hand	Chronic dorsal edema; suspected factitious lymphedema	Psychiatric + conservative	Resolution with follow-up
Whitney & Jones, 1995 [4]	1	Hand	Chronic dorsal edema; peritendinous fibrosis (MRI)	Conservative	Persistent symptoms with functional limitation
Abnousi & Chou, 2008 [3]	1	Foot	Dorsal edema, fibrotic band	Surgical excision	Symptom resolution
Birman & Lee, 2012 [12]	Review	Upper limb	Factitious disorders of the extremity	Psychiatric/varied	Highlights diagnostic overlap
Collet et al., 2014 [8]	3	Hand	Chronic dorsal edema, possible factitious cases	Conservative, psychiatric	Variable outcomes
Lemmens et al., 2019 [5]	1	Hand	Chronic dorsal edema; fibrosis (biopsy)	Conservative	Persistent symptoms
Demircioğlu et al., 2021 [2]	1	Hand	Fluctuating edema; suspected factitious etiology	Psychiatric + conservative	Symptom control
Tebbaa El Hassali et al., 2024 [6]	1	Hand	Chronic indurated dorsal edema	Conservative	Stable with follow-up

**Table 2:** Functional outcomes of the present case.

Timepoint	VAS (0–10)	QuickDASH (0–100)	Grip Strength (Jamar, % vs. Contralateral)
Preoperative	7	65	40%
Early post-op (1 month)	5	50	50%
3-Month Follow-up	4	35	65%
6-Month Follow-up	2	20	75%

VAS: Visual Analog Scale; QuickDASH: Disabilities of the Arm, Shoulder and Hand questionnaire.

## Data Availability

The original contributions presented in this study are included in the article/Supplementary Material. Further inquiries can be directed to the corresponding author.

## Supplementary Materials

The following supporting information can be downloaded at: https://www.mdpi.com/article/10.3390/jpm15120586/s1, Figure S1: PRISMA flow diagram; Table S1: search strigs used in the literature review; Table S2: PRISMA checklist.

## Abbreviations

The following abbreviations are used in this manuscript:

CRPS	Complex Regional Pain Syndrome
ICD-9-CM	International Classification of Diseases, Ninth Revision, Clinical Modification
MCP	Metacarpophalangeal
MRI	Magnetic Resonance Imaging
PRISMA	Preferred Reporting Items for Systematic Reviews and Meta-Analyses
VAS	Visual Analog Scale
