# Supplementary material for: Secretan’s Syndrome of the Hand: Literature Review and Surgical Case Report of a Rarely Documented Condition
Source: J Pers Med. 2025 Dec 1;15(12):586. doi: 10.3390/jpm15120586 (PMC12733787; doi:10.3390/jpm15120586)
Supplement: Supplementary file 1 [file jpm-15-00586-s001.zip › PRISMA 2020 flow diagram (Figure S1).pdf]

PRISMA 2020 flow diagram for new systematic reviews which included searches of databases and registers only

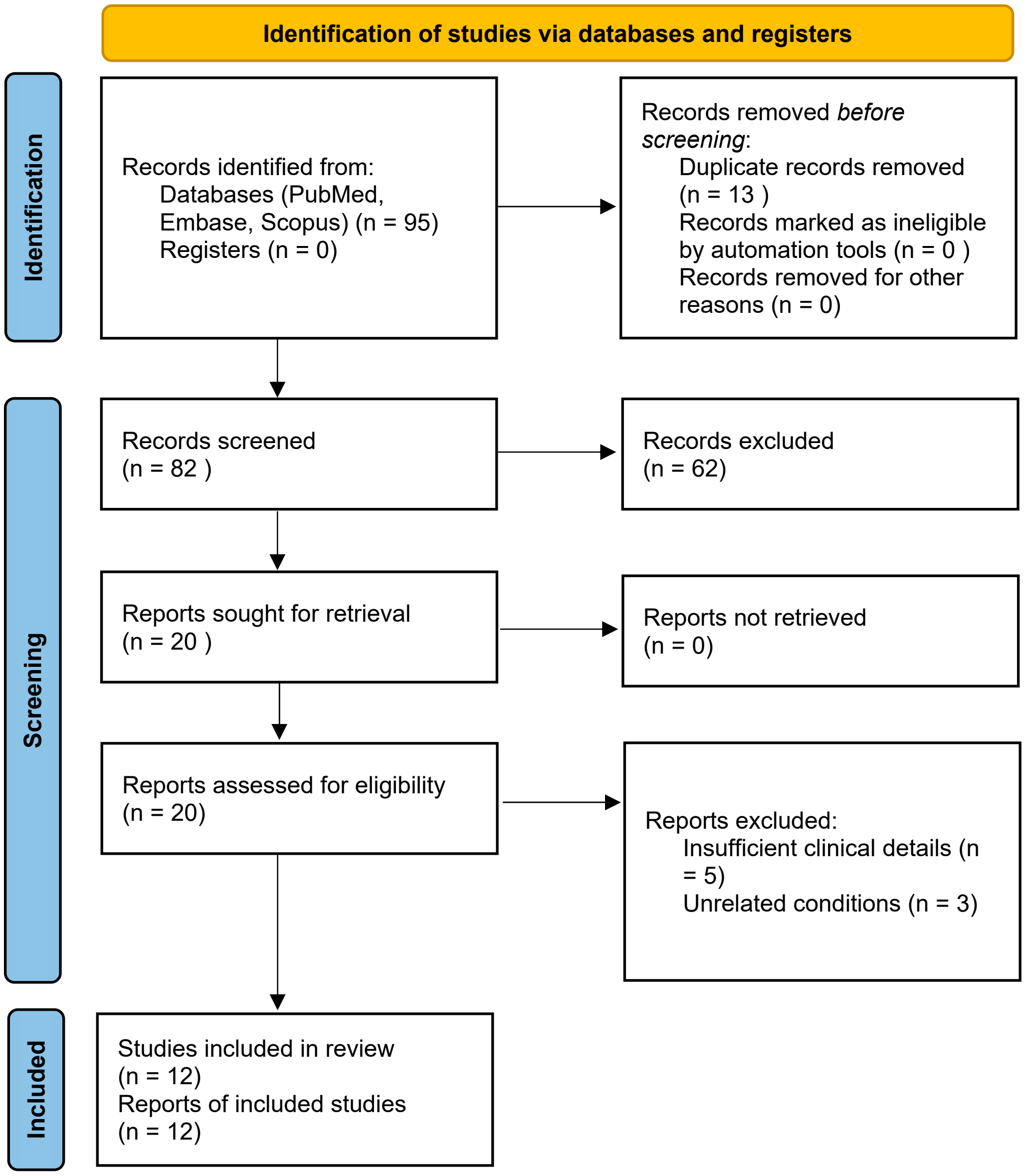

Source: Page MJ, et al. BMJ 2021;372:n71. doi: 10.1136/bmj.n71.

This work is licensed under CC BY 4.0. To view a copy of this license, visit https://creativecommons.org/licenses/by/4.0/
