# Supplementary material for: Secretan’s Syndrome of the Hand: Literature Review and Surgical Case Report of a Rarely Documented Condition
Source: J Pers Med. 2025 Dec 1;15(12):586. doi: 10.3390/jpm15120586 (PMC12733787; doi:10.3390/jpm15120586)
Supplement: Supplementary file 1 [file jpm-15-00586-s001.zip › PRISMA checklist (Table S2).pdf]

PRISMA 2020 Checklist

Section / Topic	Item	Checklist item	Location in manuscript
TITLE	1	Identify the report as a systematic review.	Title page → 'Secretan's Syndrome of the Hand: Literature Review and Surgical Case Report…'
ABSTRACT	2	Abstract structured.	Abstract → 'A systematic literature review was conducted, in accordance with the PRISMA 2020 guidelines…'
INTRODUCTION	3	Rationale.	Intro, l.3 → 'Secretan's syndrome… rare and often under-recognized condition…'
INTRODUCTION	4	Objectives.	Intro, last line → 'We report a surgically confirmed case… and critically appraise the literature…'
METHODS	5	Eligibility criteria.	Methods, l.5 → '…included case reports, case series, and reviews… excluded articles lacking sufficient clinical detail…'
METHODS	6	Information sources.	Methods, l.1 → '…PubMed, Embase, and Scopus up to March 2025…'
METHODS	7	Search strategy.	Methods, l.2 → 'Search terms included "Secretan's syndrome", "factitious lymphedema"…'
METHODS	8	Selection process.	Methods, l.6 → '…95 records identified… 12 studies included… summarized in PRISMA flow diagram (Supplementary Figure S1).'
METHODS	9	Data collection	Methods, l.5 → '…data

		process.	extracted included clinical presentation, imaging, histology, treatment, or outcome…'
METHODS	10	Data items.	Methods, 1.5 → '…clinical presentation, imaging, histology, treatment, or outcome…'
METHODS	11	Risk of bias.	Discussion, Limitations → 'Risk of bias… could not be systematically assessed…'
METHODS	12	Effect measures.	Not applicable (no quantitative synthesis).
METHODS	13	Synthesis methods.	Methods, 1.5 → '…narrative synthesis, Table 1…'
METHODS	14	Reporting bias assessment.	Not applicable.
METHODS	15	Certainty assessment.	Not applicable.
RESULTS	16	Study selection.	Results, Table 1 + Supplementary Figure S1.
RESULTS	17	Study characteristics.	Results, Table 1.
RESULTS	18	Risk of bias in studies.	Not applicable.
RESULTS	19	Results of individual studies.	Results, Section 3 → Table 1.
RESULTS	20	Results of syntheses.	Results, Section 3 → 'Treatment strategies vary widely…'
RESULTS	21	Reporting biases.	Not applicable.
RESULTS	22	Certainty of evidence.	Not applicable.
DISCUSSION	23	Interpretation.	Discussion, 1.2 → '…the imaging-based early diagnosis prevented unnecessary empiric antibiotics…'
DISCUSSION	24	Limitations.	Discussion, Limitations → '…small number of available studies… heterogeneity… risk

			of bias not assessed.'
DISCUSSION	25	Implications.	Conclusions → '…In hyperplastic forms… fasciotomies and tenolysis… can restore function.'
OTHER INFO	26	Registration/protocol.	Methods, last line → 'No protocol was registered on PROSPERO.'
OTHER INFO	27	Support/funding.	Funding → 'This research received no external funding.'
OTHER INFO	28	Competing interests.	Conflicts → 'The authors declare no conflict of interest.'
OTHER INFO	29	Data availability.	Data Availability → 'No new data were created or analyzed in this study.'
