# Supplementary material for: Secretan’s Syndrome of the Hand: Literature Review and Surgical Case Report of a Rarely Documented Condition
Source: J Pers Med. 2025 Dec 1;15(12):586. doi: 10.3390/jpm15120586 (PMC12733787; doi:10.3390/jpm15120586)
Supplement: Supplementary file 1 [file jpm-15-00586-s001.zip › Search strings (Table S1).pdf]

Table S1

The following search strings were used:

PubMed (MEDLINE):

("Secretan syndrome"[Title/Abstract] OR "Secretan's syndrome"[Title/Abstract] OR "Secretan disease"[Title/Abstract] OR "Secretan's disease"[Title/Abstract] OR "factitious lymphedema"[Title/Abstract] OR "dorsal hand edema"[Title/Abstract] OR "peritendinous fibrosis"[Title/Abstract]) AND (hand OR upper limb OR extremity OR foot)

Embase:

('secretan syndrome':ab,ti OR 'secretan's syndrome':ab,ti OR 'secretan disease':ab,ti OR 'secretan's disease':ab,ti OR 'factitious lymphedema':ab,ti OR 'dorsal hand edema':ab,ti OR 'peritendinous fibrosis':ab,ti) AND (hand OR 'upper limb' OR extremity OR foot)

Scopus:

TITLE-ABS-KEY("Secretan syndrome" OR "Secretan's syndrome" OR "Secretan disease" OR "Secretan's disease" OR "factitious lymphedema" OR "dorsal hand edema" OR "peritendinous fibrosis") AND (hand OR "upper limb" OR extremity OR foot)
